# Supplementary material for: Exome Sequencing in Adults with Unexplained Liver Disease: Diagnostic Yield and Clinical Impact
Source: Diagnostics (Basel). 2025 Aug 11;15(16):2010. doi: 10.3390/diagnostics15162010 (PMC12385362; doi:10.3390/diagnostics15162010)
Supplement: Supplementary file 1 [file diagnostics-15-02010-s001.zip › diagnostics-3770820 -23.07.2025 supplementary File 1.pdf]

Test	Normal Reference Range	Patients with abnormal values
HBsAg	Negative	-
Anti-HbsAg	Negative	All patients are > 50 IU (vaccinated)
Anti-HbcIgG	Negative	-
Anti-HbcIgM	Negative	-
HbeAg	Negative	-
Anti-Hbe	Negative	-
HBV DNA	Undetectable	-
HCV Antibody / HCV RNA	Negative	-
HDV RNA / Anti-HDV	Negative	-
HEV RNA	Negative	-
Anti-HAV/ Anti-HAV IgM	Negative	-
Anti-CMV IgA / IgG<br>Anti-EBV IgA/ IgG<br>Anti-HSV IgA/IgG	Negative<br>Negative<br>Negative	-<br>-<br>-
ANA (antinuclear antibody)	<1/40	-
SMA (Smooth muscle antibody)	<1/40	-
LKM1 (Liver-kidney microsomal antibody)	<1/40	-
Anti-LCA	<1/40	-
Anti-SLA	<1/40	-
ANCA	<1/20	-
AMA	<1/20	-

Anti-sp100	< 20 IU	-
Anti-gp210	<20 IU	-
IgG<br>IgA<br>IgM	6-15 g/L<br>0.8-3.0 g/L<br>0.4-2.5 g/L	-
Ceruloplasmin	20–35 mg/dL	P3 = 16 mg/dL<br>P4= 19 mg/dL
24 hour urinary copper	<50 µg/24 h	-
Ferritin	40-300 ng/ml	P3=310 ng/ml
Transferrin saturation	20-45 %	-
Alpha-1 antitrypsin	80–200 mg/dL	-
Apolipoprotein-B	40-100 mg/dL	P8= <25 mg/dl
LDL	50 mg/dL-130 mg/dL	P3=190 mg/dl<br>P8= 4 mg/dl
HDL	>35 mg/dl	P8= 5 mg/dl
Triglyceride	50 mg/dL-150 mg/dL	P3=270 mg/dl<br>P8= 20 mg/dL
Hba1C	<5.7%	P3= 6.8
Glucose	<100 mg/dL	P3= 124 mg/dl

HBsAg – Hepatitis B surface antigen, Anti-HBsAg Antibody to hepatitis B surface antigen, Anti-HBc IgG - IgG antibody to hepatitis B core antigen, Anti-HBc IgM – IgM antibody to hepatitis B core antigen, HBeAg – Hepatitis B e antigen, Anti-HBe - Antibody to hepatitis B e antigen, HBV DNA – Hepatitis B virus DNA, HCV Antibody / HCV RNA – Hepatitis C virus antibody / RNA, HDV RNA / Anti-HDV – Hepatitis D virus RNA / antibody, HEV RNA – Hepatitis E virus RNA, Anti-HAV / Anti-HAV IgM – Antibody to hepatitis A virus, Anti-CMV IgA / IgG – Antibodies to cytomegalovirus, Anti-EBV IgA / IgG – Antibodies to Epstein-Barr virus, Anti-HSV IgA / IgG – Antibodies to herpes simplex virus, ANA – Antinuclear antibody, ASMA- Anti-smooth muscle antibody, Anti-LKM1 – Liver-kidney microsomal antibody type 1, Anti-LCA – Anti-liver cytosol antibody type 1, Anti-SLA – Anti-soluble liver antigen antibody, ANCA – Antineutrophil cytoplasmic antibody, AMA – Antimitochondrial antibody, Anti-sp100 / Anti-gp210 – Nuclear pore complex antibodies, IgG – Immunoglobulin G, IgA – Immunoglobulin A, IgM – Immunoglobulin M, Hba1c- Glycated hemoglobin
