# Supplementary figures and images for: Exome Sequencing in Adults with Unexplained Liver Disease: Diagnostic Yield and Clinical Impact
Source: Diagnostics (Basel). 2025 Aug 11;15(16):2010. doi: 10.3390/diagnostics15162010 (PMC12385362; doi:10.3390/diagnostics15162010)

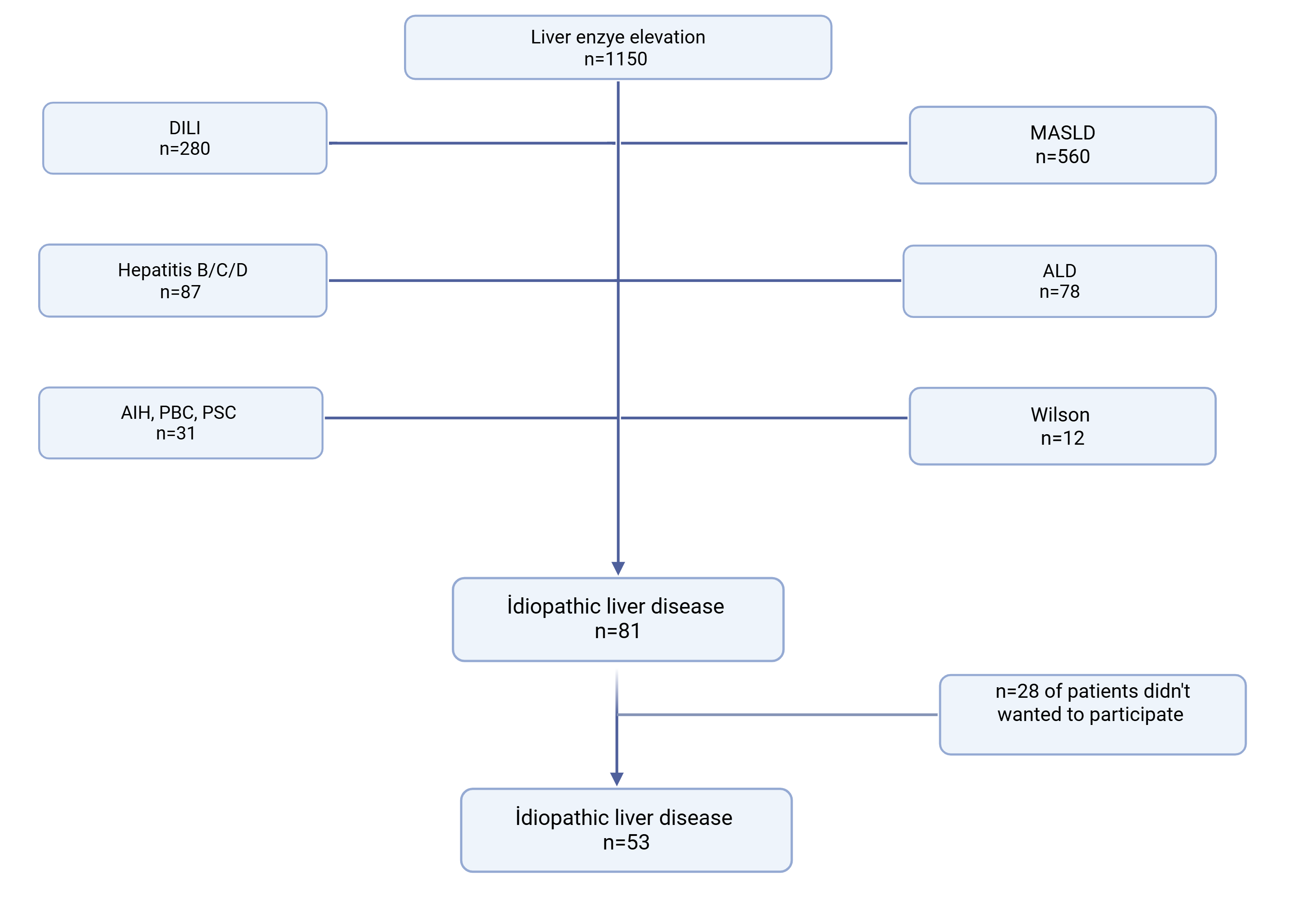

Supplement: Supplementary file 1 [file diagnostics-15-02010-s001.zip › diagnostics-3770820 -23.07.2025 supplementary file 2.png]
